# Supplementary material for: Unique advantages of dynamic l-[11C]methionine PET/CT for assessing the rate of skeletal muscle protein synthesis: A pilot trial in young men
Source: PLoS One. 2024 Jul 30;19(7):e0305620. doi: 10.1371/journal.pone.0305620 (PMC11288433; doi:10.1371/journal.pone.0305620)
Supplement: Attachment — Submitted filename: Response to Reviewers.docx [file pone.0305620.s001.docx]

April 19, 2024

Prof. Juan J. Loor,

Academic editor, ***PLOS ONE***

On behalf of all the authors, I submit our revised manuscript (Manuscript Number: PONE-D-23-43762) now titled “Unique advantages of dynamic l-[^11^C]methionine PET/CT for assessing the rate of skeletal muscle protein synthesis: a pilot trial in young men” for publication as an original article in ***PLOS ONE***.

We thank you for giving us the opportunity to revise and resubmit this manuscript. The manuscript has benefited from the insightful suggestions provided by the editors and reviewers. We have revised it as suggested, and the revised text has been indicated by red typeface for ease of review. In accordance with the comment, we revised the title and the entire manuscript regarding to description of l-[^11^C]methionine. Our point-by-point-responses to each of the comments have been provided below. We believe this revised manuscript will be of great interest to the readers of the ***PLOS ONE***. We added potential conflict of interests according to ***PLOS ONE*** statement.

This manuscript has not been published and is not under consideration for publication elsewhere. We have updated our Competing Interests Statement as follows:

“K.Y., R.N., K.T., and T.H. have no conflicts of interest to declare, but K.S., M.S., K.N., and K.A. are salaried employees of Meiji Co. However, Meiji Co. did not play any role in the study design, data collection, analysis, decision to publish, or preparation of the manuscript. This does not alter our adherence to PLOS ONE policies on sharing data and materials.”

Upon publication, we plan to upload our data to the Zenodo public repository with the Digital Object Identifier (DOI) “10.5281/zenodo.10993747.”

We appreciate your reconsideration of our manuscript for publication in ***PLOS ONE***.

Sincerely yours,

Kana Yamazaki, and on behalf of all authors

Department of Molecular Imaging and Theranostics, Institute for Quantum Medical Science, Quantum Life and Medical Science Directorate, National Institutes for Quantum Science and Technology

Email: [yamazaki.kana@qst.go.jp](mailto:yamazaki.kana@qst.go.jp)

Point-by-point responses to reviewer’s comments

Reviewer #1

The aim of the present manuscript is to evaluate the muscle protein synthesis (MPS) using dynamic 11C-methionine PET/CT in 6 healthy subjects. The study may be of interest to readers, even though the small sample size may reduce the reproducibility and the accuracy of these data.
Aside from a general improvement in the English language, the papers lacks a paragraph describing the limitations of the study, which I suggest including before considering it suitable for publication in PLOS.

We thank the reviewer for their constructive feedback regarding our manuscript. We appreciate the opportunity to enhance the clarity and impact of our work based on the reviewer’s suggestions.

We acknowledge the concern regarding the small sample size of our study, as highlighted in the comments. We understand that this may raise questions about the reproducibility and accuracy of our data. In response to the reviewer’s suggestion, we incorporated a new paragraph specifically addressing the limitations of our study, including the small sample size and its potential implications on the generalizability of our findings.

[page 17, line 391­page 18, line 394]

“Our study has limitations. Because this was a pilot study with a small sample size, we should validate our findings in a larger sample size to improve the methods for a full-scale experiment. As a next step, it would be beneficial to conduct further studies with a larger sample size or under various circumstances to evaluate *K*_i_ change more accurately.”

We believe that openly discussing these limitations will provide readers with a more comprehensive understanding of the context of our study and the cautious interpretation of its results.

We also added following descriptions that related to comments by reviewer 2.

**-Concerns regarding the time-scale of the PET/CT study**

[page 15, line 336­line 339]

“The primary objective of this study was to investigate MPS in living humans; therefore, the selected time-scale seemed appropriate and reasonable based on existing research and the physiological context of our investigation. However, further validation is required to confirm the time-scale in future studies.”

Additionally, we are committed to improving the English language quality throughout the manuscript to ensure that our findings are communicated clearly and effectively. We engaged a professional language editing service to refine our manuscript and address any linguistic issues.

We are grateful for the opportunity to revise our manuscript and believe that these changes will significantly strengthen our submission. We look forward to the opportunity of contributing to PLOS ONE.

Reviewer #2

I really understand that authors’ PET studies are very important and unique from the viewpoint of medical trials for establishing non-invasive quantitative analysis of skeletal muscle protein synthesis in men. Authors’ research strategy using the radiotracer [11C]methionine can be apprecited as a suitable approach by medical scientist because methionine serves as the initiating amino acid in the process of protein synthesis.

We greatly appreciate the positive feedback from the reviewer regarding the importance and uniqueness of our PET study in the noninvasive quantitative analysis of skeletal muscle protein synthesis. We are encouraged by the reviewer’s recognition of the importance of our research strategy and the use of [^11^C]methionine as a radiotracer.

We agree with the reviewer that the choice of methionine, being the initiating amino acid in the process of protein synthesis, makes [^11^C]methionine an appropriate radiotracer for our study objectives. This selection is grounded in solid scientific rationale, aiming to provide a more accurate and direct assessment of muscle protein synthesis, which is crucial for the understanding and management of various metabolic and muscular conditions.

However, from the general scientific view, I think that authors’ work is still in a preliminary stage and lack of scientific originality. Actually, authors did not obtain the result of significant increase in Ki after milk protein ingestion. There is less information about the quality control of the [11C]methionine injection solution such as purity, volume, and molar activity. Further, there are some concerns about the start-time of in vivo muscle protein synthesis and its time-scale of PET scan with [11C]methionine after milk protein ingestion.

Some other medical groups try to perform similar studies of some biological functions like in vivo protein synthesis using [11C]methionine which is a conventional radiotracer. If considered another possibility of the trial, parallel use of radioactive [11C]methionine and [11C]luecine might be encouraged for the assessment, the latter of which is an activator of the mammalian target of rapamycin in association with the protein synthesis.

From the comprehensive standpoint, I do not think that this article meets the journal quality and philosophy of PLOS ONE, which is a relative leading-edge journal. It might be better that this article would be summarized as a letter/communication style and submitted to more specific journals regarding PET clinical study.

Below, we address the key concerns raised and outline our steps toward improvement.

**1. Preliminary nature and scientific originality:**

We understand the reviewer’s concern regarding the preliminary stage of our work and its perceived lack of originality.

In our study, the thigh muscles were evaluated noninvasively without biopsy. This is considered one of the novel aspects.

We added the following sentences on page 13, line 283­line 285:

“The noninvasive evaluation of thigh muscles is an important advantage, because it prioritizes the comfort and safety of individuals by avoiding the need for biopsy.”

We believe our approach using [^11^C]methionine PET/CT for assessing muscle protein synthesis in humans following milk protein ingestion, offers unique insights in that we have found the potential to perform the test noninvasively and apply it to medical and other applications, despite the absence of a significant increase in *K*_i_. To improve our results, we will plan to conduct further research with larger sample sizes. Please kindly allow us to add the following descriptions:

[page 17, line 388­line 390]

“Given these aspects, our approach using [^11^C]Met PET/CT to assess MPS in humans offers unique insights, despite the absence of a significant increase in *K*_i_.”

[page 18, line 393­line 394]

“As a next step, it would be beneficial to conduct further studies with a larger sample size or under various circumstances to evaluate *K*_i_ change more accurately.”

**2. Quality control of [^11^C]methionine injection solution:**

We acknowledge the omission of detailed information on the quality control of the [^11^C]methionine injection solution. To rectify this, we included additional data on the purity, volume, and molar activity of the solution, to ensure the transparency and reproducibility of our methodology. We have added the following explanation, highlighted in red fond, to the **Materials and methods** section.

[page 7, line 139­line 140]

Radiochemical purity of the [^11^C]Met exceeded 99% and molecular activity was 162.2 ± 77.6 GBq/μmol.

[page 7, line 147]

According to a previous study (2), 6.5–7.9 MBq/kg [^11^C]Met in saline solution (484.1 ± 46.2 MBq) was administered intravenously as a bolus injection.

**3. Concerns regarding the start-time and time-scale of muscle protein synthesis in vivo:**

The reviewer’s concerns about the start-time of muscle protein synthesis in vivo and the time-scale of the PET scan are well-founded. In response, we will provide a more detailed rationale for our chosen time frames.

The time-scale we used in the present study was based on the previous study by Arentson-Lantz et al. This time-scale was reasonable and appropriate because it could be used with humans within the time frame in which MPS could be evaluated.

We have already described the concern regarding start-time and time-scale of PET study on page 15, line 320–line 336 in the Discussion section. Additionally, we added description as follows:

[page 15, line 336­line 339]

“The primary objective of this study was to investigate MPS in living humans; therefore, the selected time-scale seemed appropriate and reasonable based on existing research and the physiological context of our investigation. However, further validation is required to confirm the time-scale in future studies.”

**4. Comparison with similar studies and use of [^11^C]methionine:**

We appreciate the suggestion to compare our study with those employing [^11^C]methionine and potentially include [^11^C]leucine as a parallel radiotracer. We will review recent studies for relevant comparisons and discuss the implications of incorporating [^11^C]leucine in future research, given its role as an activator of the mammalian target of rapamycin associated with protein synthesis.

l-[^11^C]methionine is a clinically proven radiotracer with established utility in evaluating amino acid kinetics in vivo and detecting tumors. The robust application experience and ease of use of [^11^C]Met make it a valuable asset for clinical research purposes. Moreover, activity of skeletal muscle methionine adenosyltransferase, which is responsible for methionine metabolism, is 30-fold lower compared with other highly active sites such as the liver, suggests that most of the radioactivity retained in skeletal muscle after [^11^C]Met is indicative of its incorporation into skeletal muscle proteins. This characteristic enhances the possibility of performing accurate quantitative analyses of muscle protein metabolism in human subjects.

While l-[^11^C]leucine has shown effectiveness in evaluating protein synthesis rates, particularly in the brain, its use can cause complications due to the generation of ^11^CO_2_-derived radioactivity during leucine metabolism, especially in highly active skeletal muscle. Consequently, the Patlak plot analysis used in this study for image-driven MPS estimation is not currently feasible, as it requires separate consideration of blood radioactivity and ^11^CO_2_-derived radioactivity.

Despite these reports, we understand that comparisons with other amino acid tracers are also important. Therefore, we would like to add the following section.

[page 15, line 340­page 16, line 346].

“When evaluating MPS with PET/CT, it may also be necessary to consider comparing [^11^C]Met with other amino acid radiotracers, such as l-[^11^C]leucine ([^11^C]Leu). However, [^11^C]Leu is metabolically active in skeletal muscle, which makes it complicated to estimate the protein synthesis rate. Previous studies have extensively used [^11^C]Leu as a radiotracer to estimate protein synthesis rates in skeletal muscle before our initial site-specific MPS estimation. Therefore, it may be beneficial to validate the accuracy of MPS evaluation using [11C]Met in the future by comparing it to the MPS evaluation using [^11^C]Leu.”

**5. Journal quality and philosophy alignment:**

We are grateful for the reviewer’s perspective on the alignment of our article with the quality and philosophy of PLOS ONE.

We appreciate the reviewer’s perspective on our article regarding the quality and philosophy of PLOS ONE. However, we believe that this study will provide valuable original research for PLOS ONE readers in their patient care and treatment approaches.

We are committed to improving our manuscript and believe these revisions will address the concerns raised, thereby enhancing its suitability for publication. We look forward to the opportunity to contribute to the scientific community and thank the reviewer and the editorial team for their constructive comments.

P.S.
According to the IUPAC nomenclature, description of L-[11C]methionine is preferred to 11C-L-methionine.

We appreciate the reviewer’s suggestion regarding nomenclature. In accordance with the IUPAC nomenclature guidelines, as pointed out, we amended our manuscript to consistently use “l-[^11^C]methionine” or “[^11^C]Met” instead of “^11^C-L-methionine” or “^11^C-Met”. We understand the importance of adhering to standard naming conventions in scientific communication and agree that this change will improve the clarity of our manuscript.
